# TomoMQA: Automated analysis program for MVCT quality assurance of helical tomotherapy

**DOI:** 10.1002/acm2.12875

**Published:** 2020-04-08

**Authors:** Min Cheol Han, Chae‐Seon Hong, Kyung Hwan Chang, Jihun Kim, Su Chul Han, Dong Wook Kim, Kwangwoo Park, Min‐Joo Kim, Han Back Shin, Se Hyung Lee, Jin Sung Kim

**Affiliations:** ^1^ Department of Radiation Oncology Yonsei University College of Medicine Seoul Korea; ^2^ Department of Radiation Oncology Yonsei Cancer Center Seoul Korea; ^3^ Department of Biomedical Engineering Research Institute of Biomedical Engineering College of Medicine The Catholic University of Korea College of Medicine Seoul Korea; ^4^ Department of Nuclear Engineering Hanyang University Seoul Korea; ^5^ Department of Radiation Oncology Bundang Jesaeng General Hospital Gyeonggi‐do Korea

**Keywords:** DICOM, MVCT, quality assurance, radixact, tomotherapy

## Abstract

**Purpose:**

In this study, we developed a simple but useful computer program, called TomoMQA, to offer an automated quality assurance for mega‐voltage computed tomography (MVCT) images generated via helical tomotherapy.

**Methods:**

TomoMQA is written in MATLAB and contains three steps for analysis: (a) open the DICOM dataset folder generated via helical tomotherapy (i.e., TomoTherapy^®^ and Radixact™), (b) call the baseline data for the consistency test and click the “Analysis” button (or click the “Analysis” button without the baseline data and export the results as the baseline data), and (c) print an analyzed report. The overall procedure for the QA analysis included in TomoMQA is referred from the TG‐148 recommendation. Here, the tolerances for MVCT QA were implemented from TG‐148 recommended values as default; however, it can be modified by a user manually.

**Results:**

To test the performance of the TomoMQA program, 15 MVCTs were prepared from five helical tomotherapy machines (1 of TomoTherapy^®^ HD, 2 of TomoTherapy^®^ HDA, and 2 of Radixact™) in 3 months and the QA procedures were performed using TomoMQA. From our results, the evaluation revealed that the developed program can successfully perform the MVCT QA analysis irrespective of the type of helical tomotherapy equipment.

**Conclusion:**

We successfully developed a new automated analysis program for MVCT QA of a helical tomotherapy platform, called TomoMQA. The developed program will be made freely downloadable from the TomoMQA‐dedicated website.

## 1 INTRODUCTION

Mega‐voltage computed tomography (MVCT) images in a helical tomotherapy system are routinely obtained from patients during image‐guided radiotherapy (IGRT) to reduce the setup uncertainties that may occur with patient position at the time of treatment.^1^, ^2^, ^3^, ^4^ The accuracy of IGRT depends directly on the quality of the images obtained by the helical machine. Hence, periodic assessment of the quality of MVCT images performed by the quality assurance (QA) procedures is important; AAPM also strongly recommends monthly QA procedures to guarantee MVCT image quality.^5^

For such procedures in helical machines, physicists acquire an MVCT image set from the Virtual Water™ phantom (normally called “Cheese” phantom), and check the differences with respect to the baseline image [e.g., a CT image acquired at the time of machine acceptance test procedure (ATP)].

The monthly QA items and their tolerance limits for MVCT are listed in the AAPM TG‐148 report^5^ and the vendor’s manual,^6^ but unfortunately, the reports do not provide specific methods for the analysis of MVCT QA. The unconstrained analysis method might be subjective depending on the analyzer (conventionally a medical physicist), and it may require considerable time to distinguish between right and wrong. Even if several third‐party software for QA in tomotherapy are introduced in the TG‐148 report for the MVCT QA analysis,^7^ there is currently no automatic analysis tool for MVCT QA in helical tomotherapy.

In this study, we developed a simple but useful computer program, called TomoMQA, to offer automated analysis QA of MVCT images generated via helical tomotherapy, including not only TomoTherapy® (Accuray, Sunnyvale, USA) but also Radixact™ (Accuray, Sunnyvale, USA) which is a relatively new modality in radiation oncology. The program has been compiled within MATLAB (The Mathworks, Inc., Natick, MA) with a GUI interface, and to analyze MVCT QA, the program requires only two inputs — the DICOM image folder exported from tomotherapy and a previous result as baseline data.

## 2 MATERIALS AND METHODS

### 2.A Cheese Phantoms and CT acquisition

For MVCT QA, helical tomotherapy users typically utilize a cylindrical Virtual Water™ phantom (Gammex RMI, Middleton, WI), called a “Cheese” phantom, supplied by the vendor. The phantom has a diameter of 30 cm, a length of 18 cm, and several chamber holes and 20 plug holes for dose measurement and CT density tests, respectively. Fiducial markers are embedded in the middle of each phantom. To scan the cheese phantom in the helical tomotherapy system, TG‐148 and the vendor recommend setting the mode of image scanning to “fine”, that is, a slice thickness of 1 mm for SRS/SBRT (or 2 mm for non‐SRS/SBRT). The Hounsfield units (HU) data of the cheese phantom range from − 1024 corresponding to a density of zero to> 1000 corresponding to a density of the fiducial markers.

**Fig. 1:**
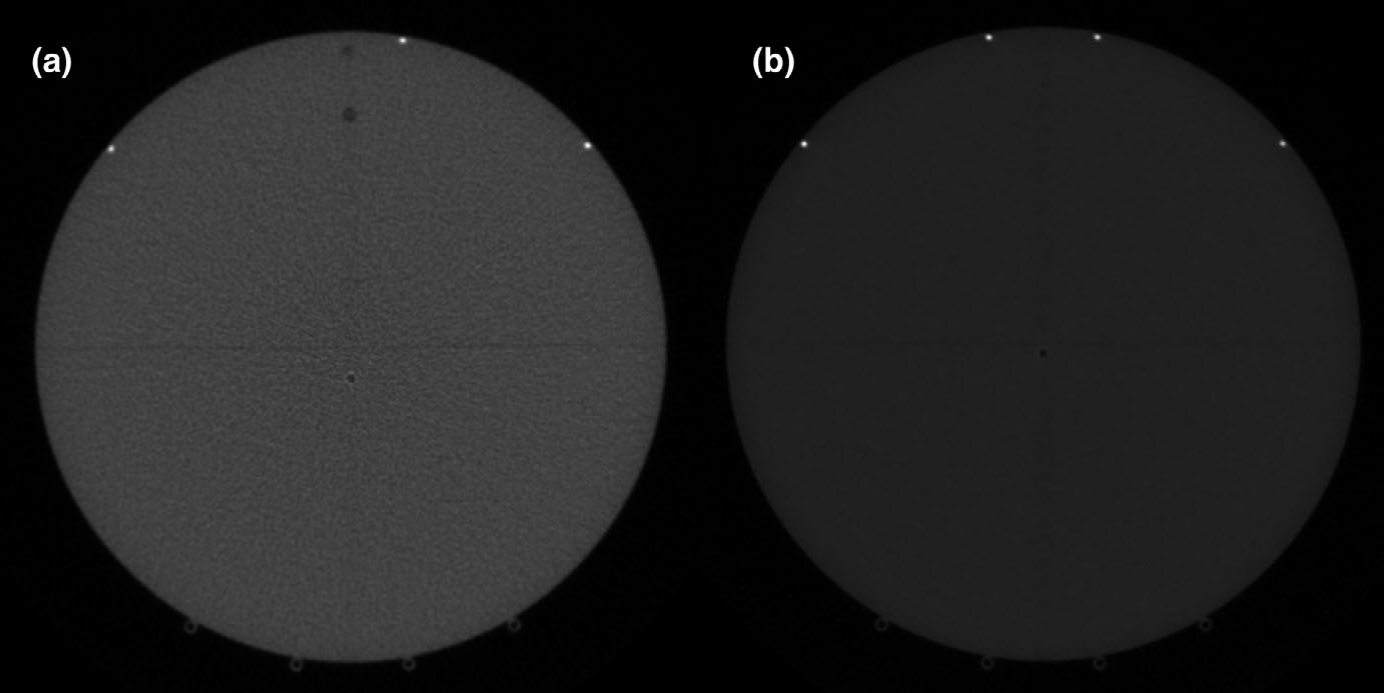
Two versions of Cheese phantom for (a) TomoTherapy^®^ HD (or HDA) and (b) Radixact™

In general, two versions of cheese phantoms are utilized for QA of helical tomotherapy; there is no difference between them, except for body color and the number of fiducial markers embedded in the phantoms. Color dose does not affect any analysis of MVCT QA; however, fiducial markers are displayed differently, as shown in Fig. 1.

### 2.B Mechanism of TomoMQA

TomoMQA is written in MATLAB and is created as a graphical user interface (GUI) to facilitate easy utilization for users who need to examine the QA of MVCT in helical tomotherapy. Before QA analysis, TomoMQA reads an MVCT image dataset in DICOM format. In general, the DICOM dataset comprises several DICOM files that contain image data and various attributes that identify the metadata such as position, series number, image resolution, and pixel slice thickness. In addition, each DICOM file contains a two‐dimensional (2D) HU matrix to represent each slice image. In TomoMQA, the images are automatically sorted based on InstanceNumber and reconstructed as a three‐dimensional (3D) matrix array in memory based on PixelSpacing, SliceThickness, and HU matrix data.

For the analysis of MVCT QA, TomoMQA can evaluate the following four monthly QA items: (a) geometric distortions, (b) uniformity and noise, (c) contrast, and (d) spatial resolution. Pertinent image slices for each analysis are selected from the 3D array, and detailed information is provided in the following subsections.

#### 2.B.1 Consistency test for geometric distortions

To test the geometric distortions of the MVCT, the dimensions in the x‐ and z‐directions (i.e., transaxial plane) should be measured from the distances between the markers. In addition, the longitudinal direction (i.e., y‐direction) should be measured from the distances between a marker and the surface of the cheese phantom. The distances between the measured lengths in two MVCT images (i.e., axial and coronal images) and the physical lengths of the phantom should be compared, and then the difference should be evaluated within 1 mm for SRS/SBRT (or 2 mm for non‐SRS/SBRT).

**Fig. 2:**
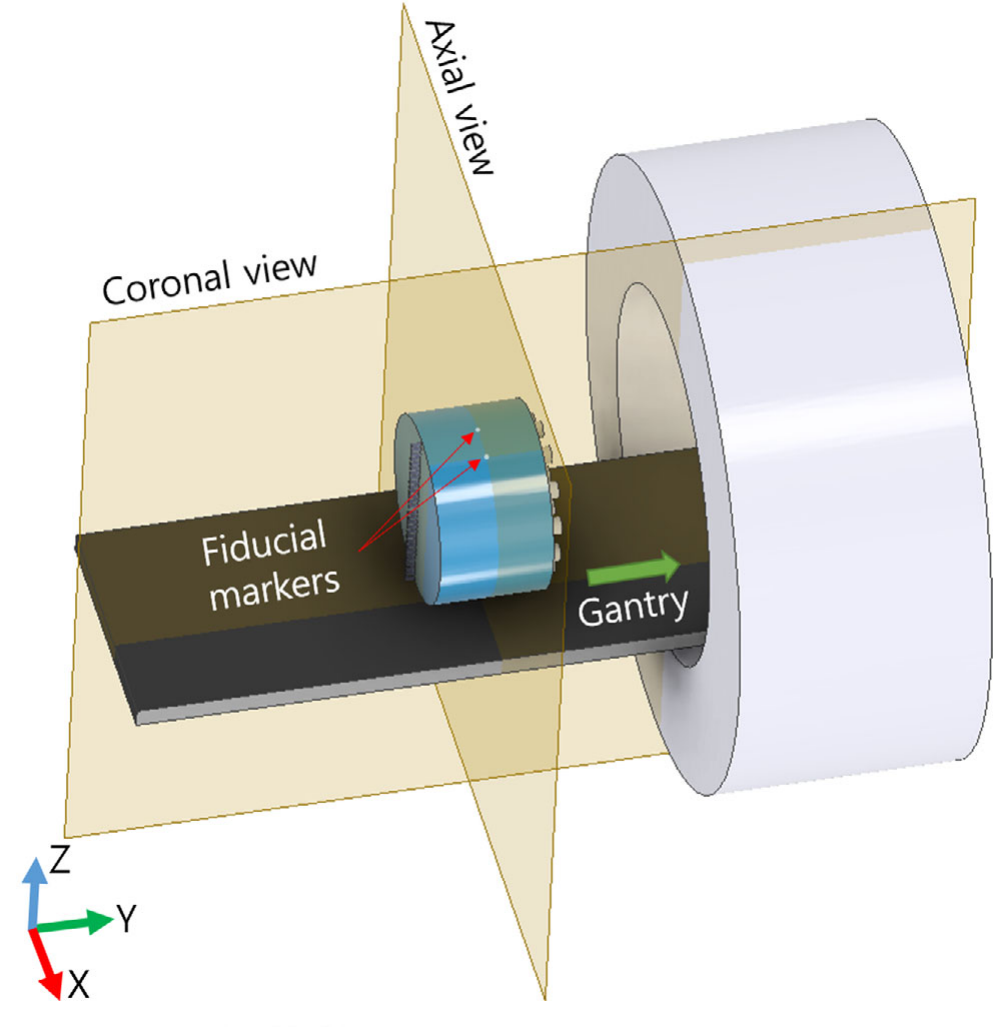
Schematic of the central views of the cheese phantom for geometric distortions

TomoMQA is designed to locate a marker‐embedded slice (i.e., the middle of the phantom) in the MVCT image dataset as a first step and to define the middle slice of the phantom. Subsequently, the selected image is converted to a binary image to distinguish the position of the markers clearly, and the centroid of each fiducial marker is determined by using the *regionprops* function implemented in MATLAB.^8^ Finally, the distances for the x‐ and z‐directions in images are calculated between the markers, and the longitudinal distance is also calculated between one of the markers and a boundary of the phantom. The calculated results are compared to the baseline data. Figure 2 depicts the schematic design of the image slices selected via TomoMQA for the geometric distortion test.

All results are automatically calculated and displayed in figures included in TomoMQA. Note that TomoMQA recognizes only three markers to ensure the high compatibility of the two different types of phantoms; hence, even if the four fiducial markers are embedded in the phantom, one of the markers will be ignored during distance calculation. For convenience, a user can select either SRS/SBRT or non‐SRS/SBRT with an option box.

#### 2.B.2 Consistency test for uniformity and noise

When assessing image uniformity, the average HU in a small region of interest (ROI) (e.g., a circle of approximately 10‐mm diameter) located at the center of a specific image should be calculated and compared to ROIs located in the periphery. Subsequently, the largest difference between the HUs of the center and periphery ROIs should be calculated. If an MVCT image is used for dose calculation, TG‐148 recommends that the difference should be < 25 HU. When assessing the image noise, the standard deviations (σCT) of the HUs in the central ROIs should be calculated. TG‐148 mentioned that the noise levels (i.e., one standard deviation) are typically around 50–70 HU; however, the detailed information, such as an area of each ROIs, is not described in TG‐148.

**Fig. 3:**
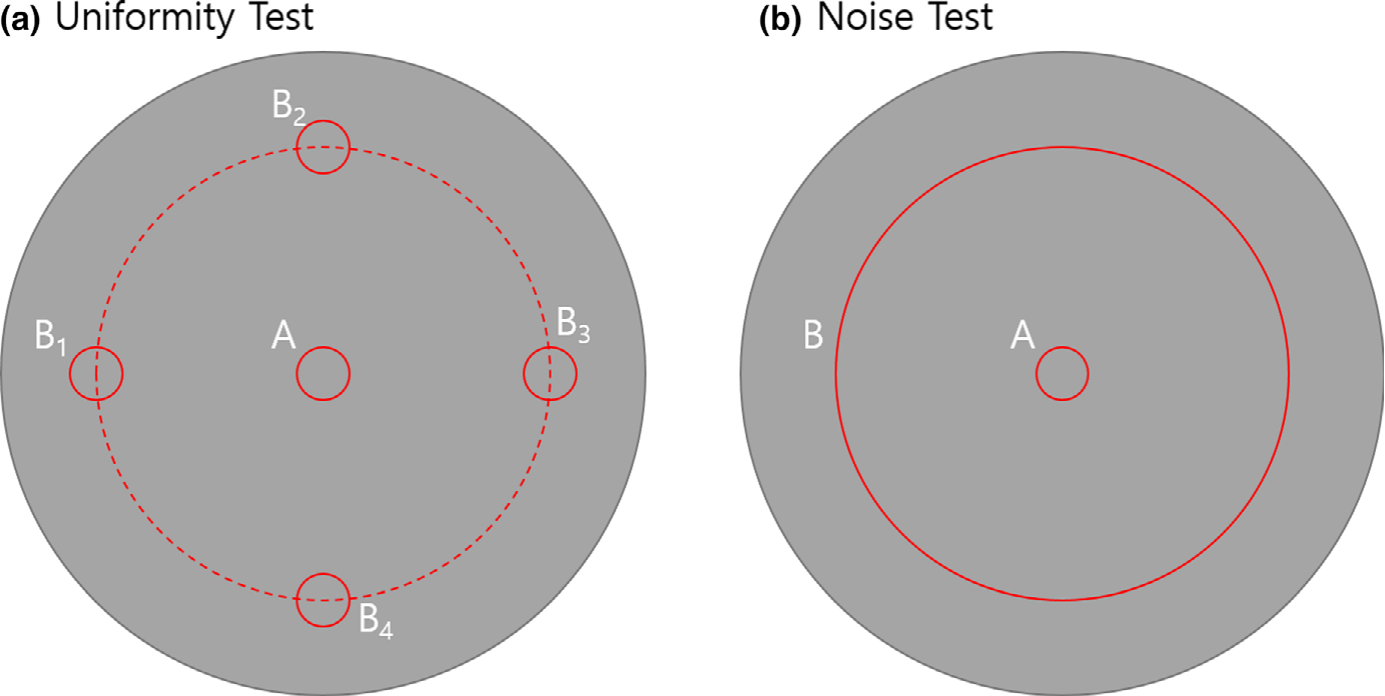
Schematic of region of interests used in TomoMQA for uniformity and noise tests

TomoMQA is designed such that it can select the pertinent image slice that contains a uniform section to assess the uniformity and noise of MVCT images. Typically, the uniform slice of the cheese phantom is located between the middle of the phantom and the edge of the A1SL chamber holes (approximately 25 mm thickness); hence, in here, the TomoMQA selects the slice 10 mm from the middle of the phantom. Regarding the uniformity test, a total of five circle‐type ROIs are created from a center point and four cardinal points on the selected slice for the uniformity test. Subsequently, the average HU is calculated from each ROI, and the largest difference between the central HU and other HUs is determined to assess the uniformity of MVCT images. For the noise test, a big ROI located in the center of the phantom is additionally created, and the standard deviations (σCTs) are calculated for the small and big ROIs located in the center. Figure 3 shows the schematic of ROIs used in TomoMQA for uniformity and noise tests.

Unfortunately, regarding these two tests, the vendor and TG‐148 recommendations do not include several test‐related parameters (e.g., distance from center to the periphery site, ROI size for noise test). Especially, the value of tolerance limits for the uniformity test is only suggested when the MVCT image is used for dose calculation. In TomoMQA, as a default, the tolerance limit of the uniformity test for only imaging usage was set as equal to the reference for other materials used in the contrast evaluation (Section 2.B.3). For convenience, in TomoMQA, the user can control the related parameters (e.g., ROI size, distance from center to periphery ROI, and tolerance limit) on GUI. The results of the uniformity and noise tests calculated using TomoMQA are not compared with the baseline data, because the uniformity and noise values are inherent characteristics of their own images; baseline data are printed in a QA report solely for reference.

#### 2.B.3 Consistency test for contrast

**Fig. 4:**
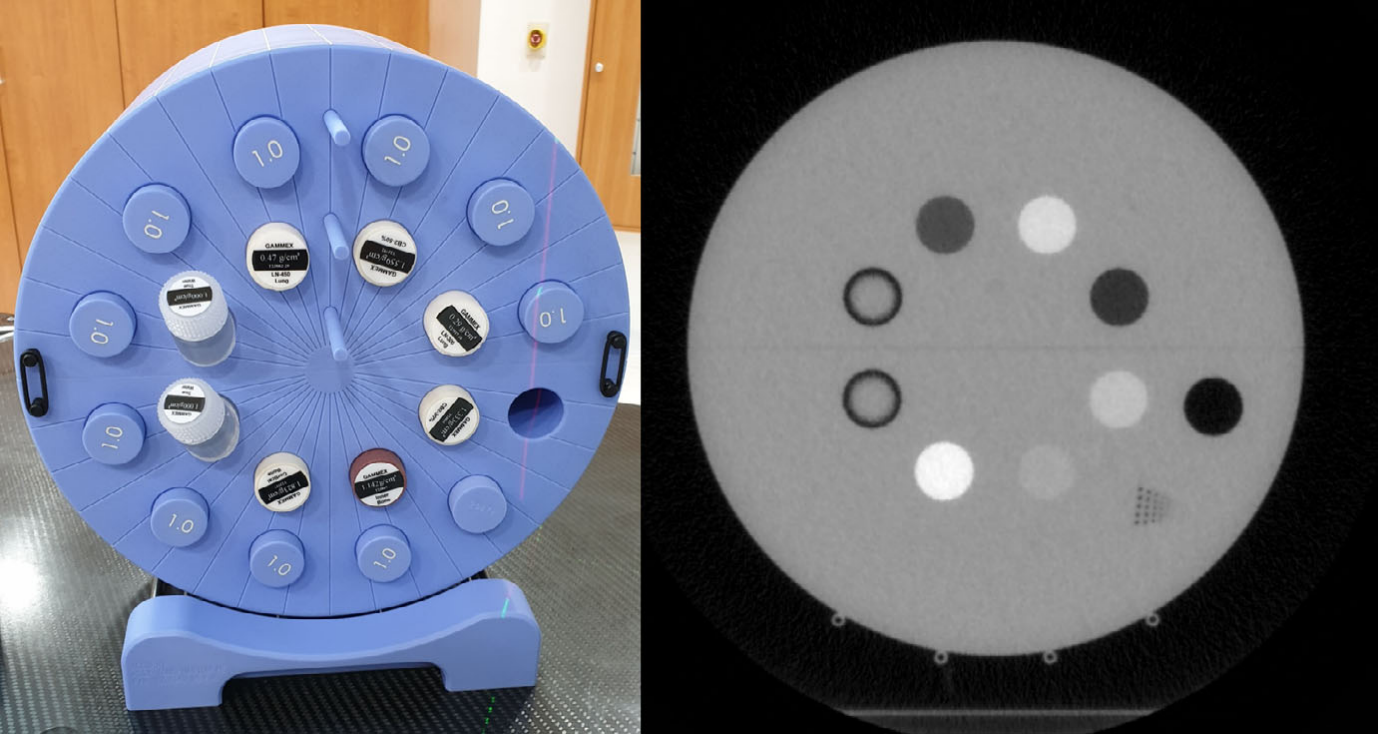
View of the cheese phantom (left) and its mega‐voltage computed tomography image (right). The various material plugs and high‐contrast resolution plug are inserted as shown in the photo, and one of holes is ejected to measure an air density

The test for the image contrast should be conducted by inserting various density plugs supplied by the vendor. Figure 4 shows the cheese phantom with various density plugs inserted (left) and its corresponding MVCT image (right). For the assessment of image contrast, the average HUs of the density plugs should be measured and compared to baseline data. The TG‐148 recommends that if the MVCT dataset is used for dose calculations, the average HUs calculated from the density plugs are within 30 and 50 HU deviation from baseline data. Unfortunately, if the MVCT dataset is not used for dose calculations, the vendor and TG‐148 recommendations do not provide acceptable tolerance limits for the evaluation.

**Fig. 5:**
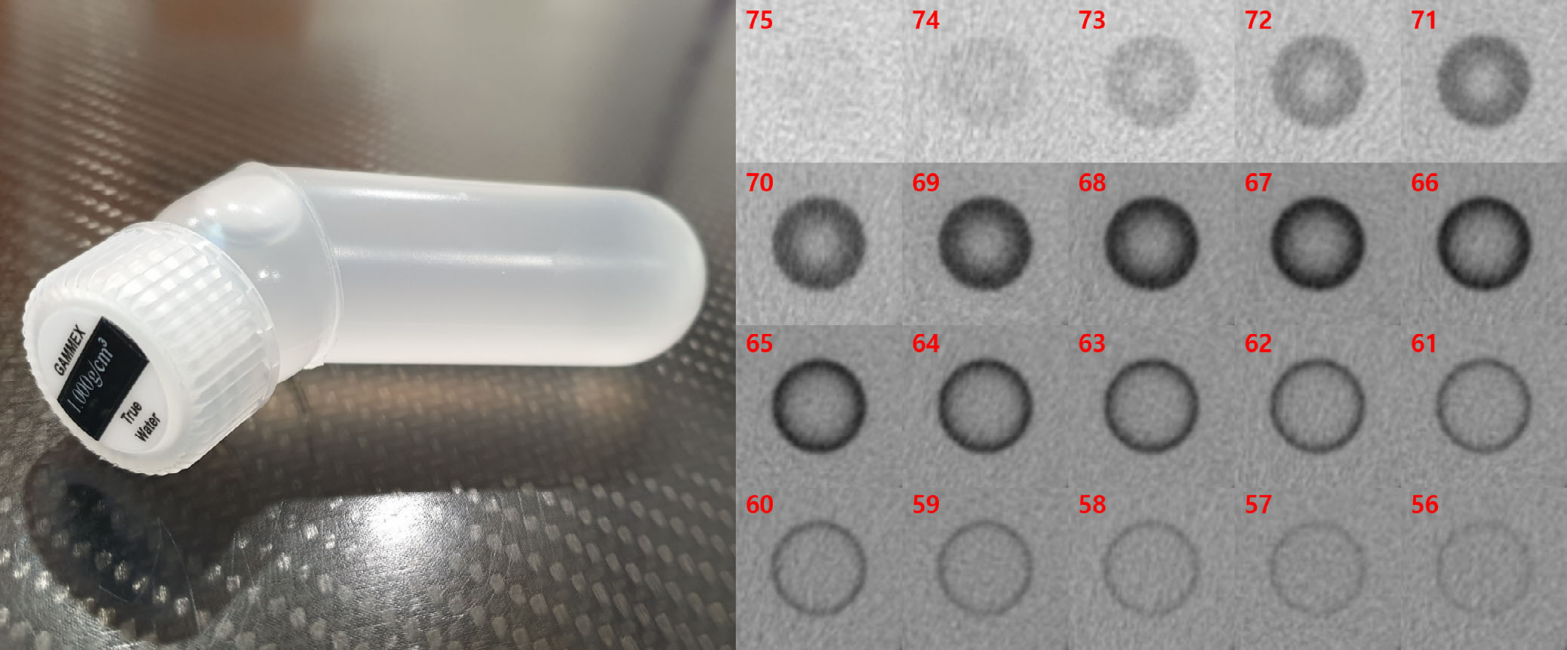
Liquid‐type container for true water (Gammex RMI, Middleton, WI) (left) and axial computed tomography images of the true water container according to slice number (right)

TomoMQA is designed to assess the contrast quality based on the pertinent slice located ~ 50 mm from the middle slice to the edge of the cheese phantom; the distance from the middle of the phantom and the end of plug‐in hole is approximately 30 mm; however, an additional ~ 20‐mm depth is required to measure the liquid‐type plugs (i.e., a true water container) as shown in Fig. 5.

Typically, the plug holes are positioned at the fixed location for either type of phantom; hence, the average HU of the specific area for each plug can be calculated easily if the center point of the phantom is known. In TomoMQA, the size of ROI to calculate the average HU is considered to be same as in the uniformity test. For the analysis, each calculated HU is compared with the corresponding value in the baseline that is close to the calculated HU irrespective of a plug position, and whether its error is consistently in the tolerance limits of the user is verified. All results are automatically calculated and are displayed in a table included in TomoMQA, and comparison targets (i.e., plugs) can be selected by a user in GUI.

#### 2.B.4 Test for spatial resolution

The test for the spatial resolution should be performed by inserting the high‐contrast resolution plug supplied by the vendor. TG‐148 recommends that the minimum resolution of 1.6 mm should be visible in the reconstructed CT images. Note that total 7‐type holes (i.e., 0.8, 1.0, 1.2, 1.4, 1.6, 1.8, and 2.0 mm in diameter) are on the surface of the resolution plug, and the depth of the 1.6‐mm holes is 5 mm in minimum. Typically, the spatial resolution has been nominally assessed by a visual inspector.

TomoMQA is designed to assess the spatial resolution of the MVCT image based on the pertinent slice located 35 mm or 90 mm from the middle slice depending on the insert direction of the plug. In TomoMQA, the desired image with the plug hole can be identified automatically, because the spatial resolution plug generally has a maximum noise value among the inserted plug‐in due to its inhomogeneous structure. If the TomoMQA could not identify the plug automatically (e.g., owing to the image artifact), a user can vary the number of the resolution plugs manually in the GUI.

In TomoMQA, the image of resolution plug is detected and displayed in a figure, and its window level is also automatically set considering the HU range of the image. The analysis with respect to the spatial resolution in TomoMQA is designed as an exception to be manually conducted by a user. In practice, in contrast with the previous evaluations, the resolution assessment completely depends on the eye of the inspector.

#### 2.B.5 Generation of Report for MVCT QA

After completing the analysis of MVCT, TomoMQA can print out the MVCT QA report in a pdf format. The report comprises only one page and includes analysis metadata such as analysis results, baseline results, grades of assessment items (i.e., pass/fail), and a screenshot of TomoMQA program.

## 3 RESULTS AND DISCUSSION

### 3.A TomoMQA

**Fig. 6:**
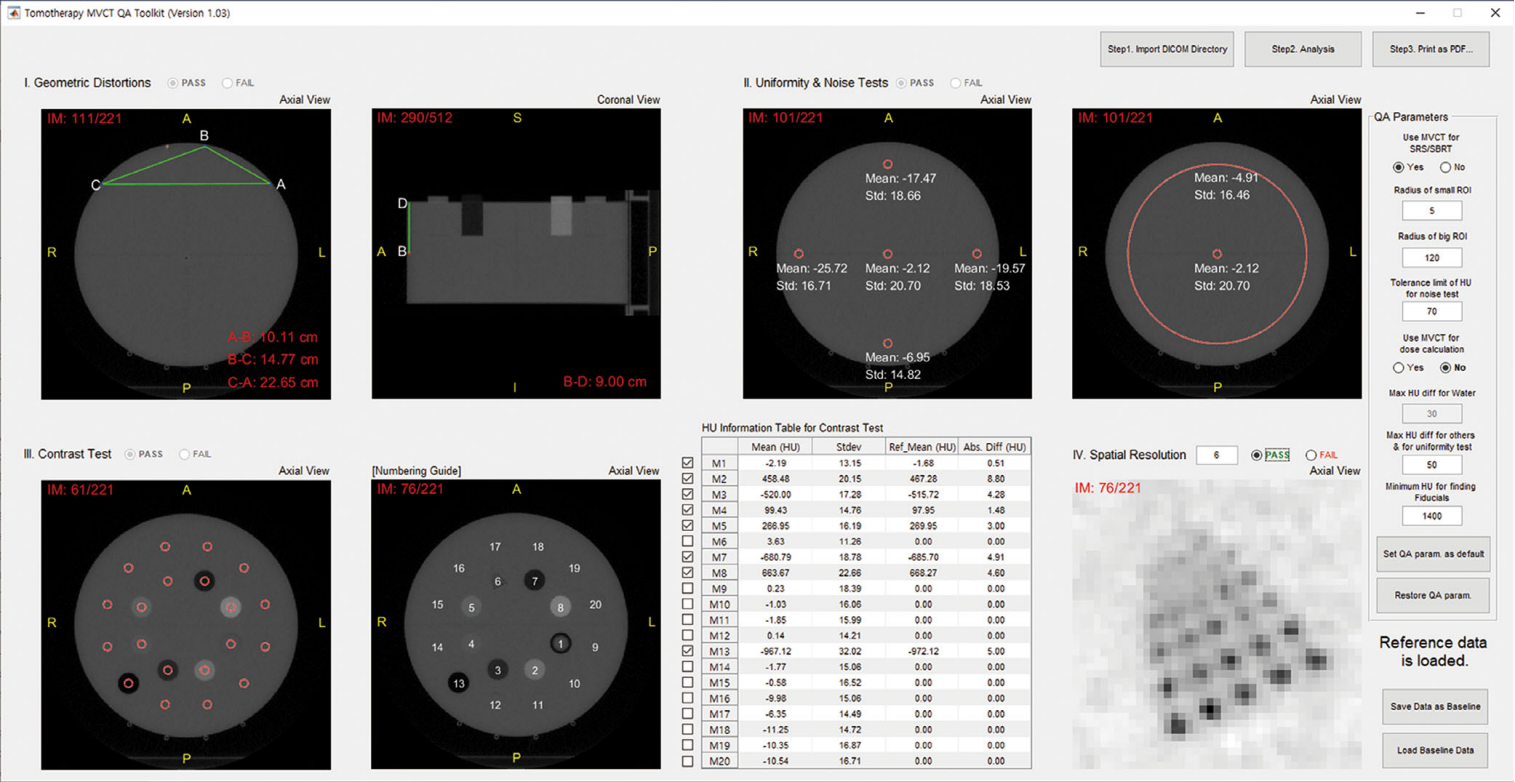
Screenshot of TomoMQA graphical user interface

**Fig. 7:**
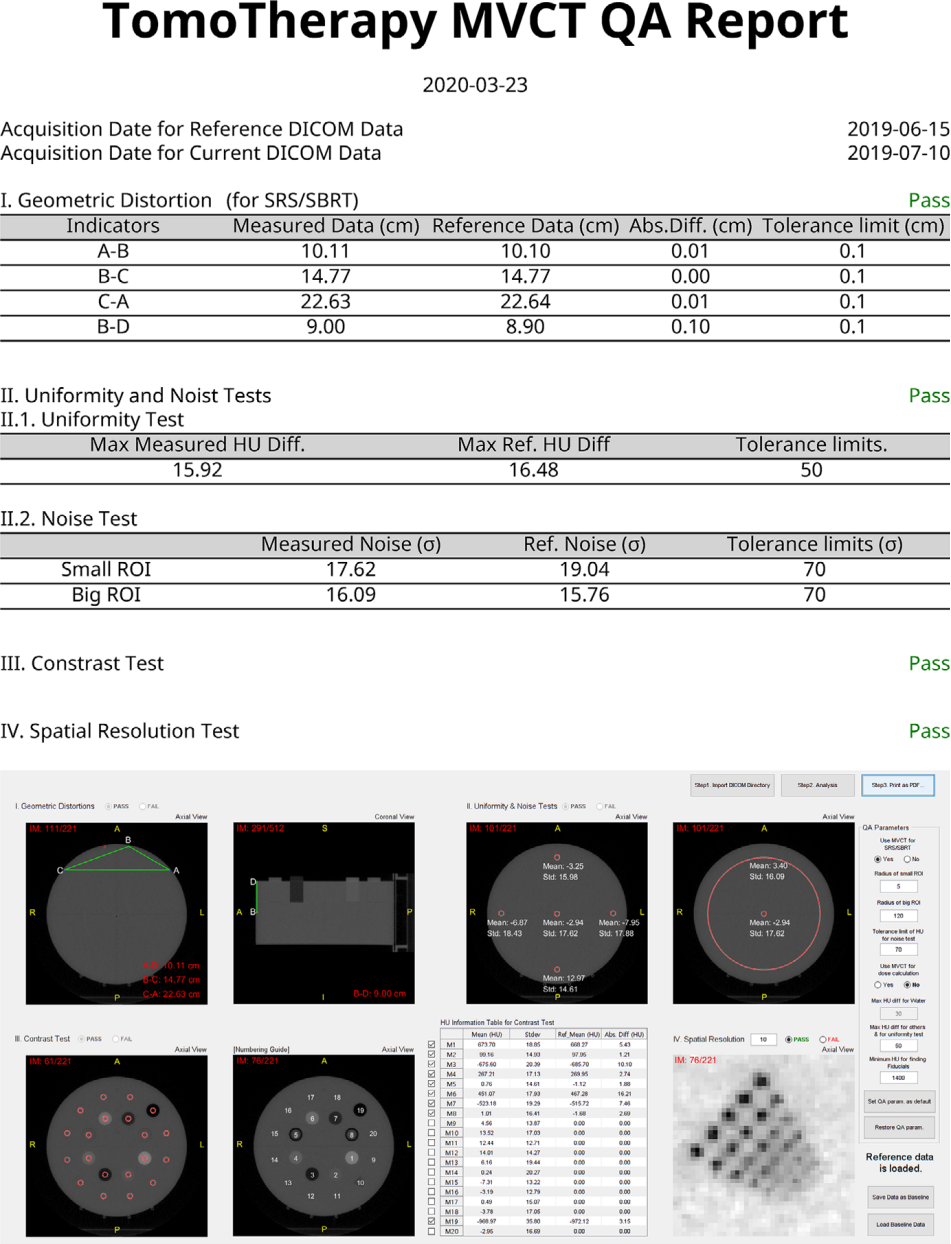
Example of mega‐voltage computed tomography quality assurance report written by TomoMQA, The report includes all results with the corresponded reference data and the screenshot of TomoMQA graphical user interface

Figure 6 shows an example of MVCT QA analysis using TomoMQA. The initialization procedures are automatically conducted when a DICOM dataset is imported, and all analyses are initiated after clicking the analysis button. Run‐times of the program were around ten seconds for importing each of the ~ 220 DICOM images (total ~ 110 MB), and a few seconds for analysis. As shown in the figure, QA‐related parameters (e.g., circle radius, tolerance limit, and HU difference limits) are modified and a user can save new parameters by default. The analysis results can be also saved as baseline data. The animation for complete demonstration of the TomoMQA is available in Ref. [9]. Figure 7 shows an example of the analyzed MVCT QA report. The report includes all results with the compared reference data and the screenshot of TomoMQA GUI for users convenience. As shown in the figure, TomoMQA visually shows that the analytical process and report functionality were successfully performed.

To evaluate the performance of the developed program, several MVCT images were acquired by a total of five helical tomotherapy machines (i.e., 1 of Tomotherapy^®^ HD, 2 of Tomotherapy^®^ HDA, and 2 of Radixact™) for three months, and the image sets were evaluated using TomoMQA repeatedly. From all MVCT images, TomoMQA found the fiducial markers embedded in two types of cheese phantoms, irrespective of any rotations of cheese phantoms (i.e., 0°, 90°, 180°, and 270°). The pertinent slices for the QA analysis were also automatically detected in TomoMQA. Furthermore, the program recognized the correct position of spatial resolution plug from a total of 15 MVCT image sets, irrespective of the inserted direction of the resolution plug. Finally, TomoMQA provided consistent analysis results when repeating analysis procedures for the same MVCT images, in contrast with those of manual analysis. From our evaluations, consequently, it was confirmed that TomoMQA can successfully perform the MVCT QA analysis irrespective of a type of helical tomotherapy equipment.

### 3.B Example QA by using TomoMQA

For instance, Table 1 summarizes the example results of TomoMQA to evaluate the specific MVCT sets acquired for the check of machine issues as follows:

- QA #1: artifact issue
- QA #2: after fixing artifact issue
- QA #3: after replacement of the MVCT detector

**Table 1:** Analysis examples of mega‐voltage computed tomography quality assurance (QA) by using TomoMQA

Events	Geometric distortions (cm)				Uniformity (HU)	Noise (HU)	Contrast (HU)							Spatial resolution
	A–B	B–C	C–A	B–D			LN‐300	LN‐450	Water	Inner bone	CB2‐30%	CB2‐50%	Cortical bone	
Baseline	10.10	14.77	22.64	8.90	16.48	19.04	‐685.70	‐515.72	‐1.12	97.95	269.95	467.28	668.27	Pass
QA #1	10.13	14.76	22.66	8.90	24.78	88.18	‐690.51	‐522.24	2.26	93.87	257.99	458.58	664.90	Pass
QA #2	10.11	14.76	22.64	9.00	15.92	17.62	‐675.60	‐523.18	0.76	99.16	267.21	451.07	673.70	Pass
QA #3	10.11	14.76	22.64	9.00	13.06	17.98	‐703.38	‐538.26	‐41.84	61.51	227.09	409.13	614.14	Pass

As summarized in Table 1, the noise parameter of QA #1 increased compared with other parameters owing to the artifact issue, and subsequently, the noise parameter was returned after fixing the artifact issue (QA #2). After replacement of the MVCT detector (QA #3), TomoMQA reported that the contrast HUs of two materials (i.e., Water, CB2‐50%) acquired from a new detector were different from those of the baseline. Especially, the CT number of a water measured lower value (i.e., −41 HU); if your MVCT images are used for dose calculations, in this case, a physicist should request CT number calibration to an engineer, and then re‐generate a new baseline data in TomoMQA using “Save Data as Baseline” button.

## 4 CONCLUSION

In the present study, we developed a simple yet useful program, called TomoMQA, which can be used to analyze MVCT images generated from helical tomotherapy such as TomoTherapy^®^ and Radixact™. Our test results demonstrated that TomoMQA can successfully evaluate the quality of MVCT images, while complying with the guidelines of AAPM TG‐148. We believe that TomoMQA will be useful to analyze MVCT QA images acquired from helical tomotherapy, and it will help save time compared to manual analysis of MVCT QA measurements. The developed program can be freely downloaded from the TomoMQA‐dedicated website,^9^, ^10^ and the program will be updated to overcome current limitations (e.g., loading speed, reported bugs) continuously.

## CONFLICT OF INTEREST

The authors declare that they have no competing interests.
